# Supplementary material for: Ultrasound versus fluoroscopy as imaging guidance for percutaneous nephrolithotomy: A systematic review and meta-analysis
Source: PLoS One. 2023 Mar 2;18(3):e0276708. doi: 10.1371/journal.pone.0276708 (PMC9980746; doi:10.1371/journal.pone.0276708)
Supplement: S1 File — The search strategy for database searching. (DOCX) [file pone.0276708.s002.docx]

**Search strategies**

**PubMed:**

| #1 | (Ultrasound[Title/Abstract]) OR (US[Title/Abstract])) OR (ultrasound-guided[Title/Abstract])) OR (ultrasonography[Title/Abstract])) OR (ultrasonographic[Title/Abstract])) |
| --- | --- |
| #2 | (fluoroscopy[Title/Abstract]) OR (X-ray[Title/Abstract])) OR (fluoroscopic[Title/Abstract])) |
| #3 | (percutaneous nephrolithotomy[Title/Abstract]) OR (minimally invasive percutaneous nephrolithotomy[Title/Abstract])) OR (PCNL[Title/Abstract])) |
| #4 | **#1 AND #2 AND #3**  (((("Ultrasound"[Title/Abstract] OR "US"[Title/Abstract] OR "ultrasound-guided"[Title/Abstract] OR "ultrasonograpgy"[Title/Abstract] OR "ultrasonographic"[Title/Abstract]) AND "fluoroscopy"[Title/Abstract]) OR "X-ray"[Title/Abstract] OR "fluoroscopic"[Title/Abstract]) AND "percutaneous nephrolithotomy"[Title/Abstract]) OR "minimally invasive percutaneous nephrolithotomy"[Title/Abstract] OR "PCNL"[Title/Abstract] |

**Embase:**

| #1 | 'Ultrasound':ab,ti OR 'US':ab,ti OR 'ultrasound-guided':ab,ti OR 'ultrasonography':ab,ti OR 'ultrasonographic':ab,ti |
| --- | --- |
| #2 | 'fluoroscopy':ab,ti OR 'X-ray':ab,ti OR 'fluoroscopic':ab,ti |
| #3 | 'percutaneous nephrolithotomy':ab,ti OR 'minimally invasive percutaneous nephrolithotomy':ab,ti OR 'PCNL':ab,ti |
| #4 | **#1 AND #2 AND #3**  'Ultrasound':ab,ti OR 'US':ab,ti OR 'ultrasound-guided':ab,ti OR 'ultrasonography':ab,ti OR 'ultrasonographic':ab,ti AND 'fluoroscopy':ab,ti OR 'X-ray':ab,ti OR 'fluoroscopic':ab,ti AND 'percutaneous nephrolithotomy':ab,ti OR 'minimally invasive percutaneous nephrolithotomy':ab,ti OR 'PCNL':ab,ti |
